# Comparative ESEM Characterization and Collagen-Related Tissue Responses to Commercial Injectable Bioregenerative Formulations in a Murine Model

**DOI:** 10.3390/ijms27114936

**Published:** 2026-05-29

**Authors:** Anna Paula Silva Dias Marcondes, Fernando Veloso Caldeira Barcellos, Maria Rafaela Pereira Lacerda, Andréia Luiza Oliveira Costa, Lorena dos Reis Pereira Queiroz, Jhenifer Rocha Oliveira, Bruno Gorayski Milo, Tatiany Bertollo Cozer Ribeiro da Costa, Sérgio Henrique Sousa Santos, Lucyana Conceição Farias, Alfredo Maurício Batista de Paula, André Luiz Sena Guimarães

**Affiliations:** 1 Department of Dentistry, Universidade Estadual de Montes Claros (Unimontes), Montes Claros 39401-089, Minas Gerais, Brazil; annapsdias@gmail.com (A.P.S.D.M.); andreialuizaaa594@gmail.com (A.L.O.C.); lorenadosreis31@gmail.com (L.d.R.P.Q.);; 2 Institute of Agricultural Sciences, Universidade Federal de Minas Gerais (UFMG), Montes Claros 39404-547, Minas Gerais, Brazil

**Keywords:** poly-L-lactic acid, hyaluronic acid, polydeoxyribonucleotide, dermal remodeling, bioregenerative formulations

## Abstract

Dermal senescence is associated with reduced fibroblast activity, decreased extracellular matrix synthesis, and impaired tissue repair. Commercial injectable formulations containing poly-L-lactic acid (PLLA), hyaluronic acid (HA), and polydeoxyribonucleotide (PDRN)-associated compounds have been proposed for dermal remodeling approaches and modulate tissue-response pathways; however, comparative studies evaluating commercially available formulations under standardized experimental conditions remain limited. This study aimed to characterize the morphology of formulations containing PLLA, HA, and PDRN, used alone or in combination, by environmental scanning electron microscopy (ESEM), and to investigate formulation-associated histological and collagen-related molecular responses in a murine model. Formulations containing PLLA, HA, HA + PDRN, and PLLA, HA and PDRN were administered into the dorsal subcutaneous tissue of 14 *Mus musculus* mice (Swiss strain). After 30 days, tissue response was assessed by ultrasound, histological analysis with Masson’s trichrome staining, and RT-qPCR quantification of collagen-related gene expression. ESEM analysis revealed distinct morphological characteristics among the biomaterials, and the combined PLLA, HA and PDRN formulation exhibited a more complex and integrated multiphase structure. Histological analysis showed preserved tissue architecture in all groups, with no evidence of marked inflammatory response or structural disruption. RT-qPCR demonstrated significantly higher COL1A1 expression in the PLLA-only and PLLA, HA, and PDRN groups compared with controls (*p* < 0.05), whereas no significant differences were observed for COL2A1 or COL3A1. These findings indicate that PLLA-containing formulations were associated with selective COL1A1 upregulation under the evaluated conditions, suggesting formulation-associated collagen-related molecular responses in this short-term model.

## 1. Introduction

Dermal aging is associated with progressive alterations in extracellular matrix organization, fibroblast activity, collagen turnover, and tissue biomechanical properties, contributing to reduced structural support and impaired skin homeostasis [1,2,3,4]. These changes are frequently accompanied by dermal thinning, collagen fragmentation, extracellular matrix disorganization, reduced elastin and hyaluronic acid content, and increased activity of matrix-degrading enzymes [3]. As a result, aged dermal microenvironments may become less responsive to remodeling-related signaling and less capable of supporting efficient tissue repair [5,6,7].

Molecular studies have shown that dermal remodeling is influenced by pathways associated with extracellular matrix organization, inflammation, angiogenesis, metabolism, and collagen-related transcriptional regulation [8]. Therefore, the evaluation of injectable bioregenerative formulations may benefit from integrating structural, histological, imaging-based, and molecular analyses to better characterize tissue response patterns associated with different formulations.

Injectable biomaterial-based formulations, including poly-L-lactic acid (PLLA)-, hyaluronic acid (HA)-, and polydeoxyribonucleotide (PDRN)-containing products, have increasingly been incorporated into clinical protocols aimed at dermal remodeling and extracellular matrix support [9,10,11,12]. PLLA-containing formulations have been associated with delayed collagen-related tissue responses and progressive extracellular matrix remodeling following controlled tissue stimulation [13,14]. HA-based formulations may contribute to hydration, viscoelastic support, and extracellular matrix organization, depending on their physicochemical properties, concentration, rheological behavior, and degree of cross-linking [12,15]. Commercial formulations containing PDRN-associated compounds have also been proposed as adjunctive bioregenerative strategies. They may modulate tissue response pathways associated with angiogenesis, extracellular matrix organization, inflammation control, and cellular recovery [16]. However, when PDRN-containing products include additional bioactive compounds, their biological effects should be interpreted as formulation-associated responses rather than as effects exclusively attributable to PDRN.

Despite the increasing clinical use of injectable bioregenerative products, comparative experimental studies evaluating commercially available formulations under standardized conditions remain limited [8]. In particular, it remains unclear how PLLA-, HA-, and PDRN-containing formulations, used alone or in combination, differ in their ultrastructural characteristics, tissue distribution patterns, imaging-based behavior, histological response, and collagen-related molecular profile in short-term *in vivo* models. This represents an important gap because complex commercial formulations may not reproduce the behavior of isolated purified biomolecules, and their effects cannot necessarily be attributed to a single component.

Hydrated polymeric systems and injectable formulations may exhibit distinct porosity, surface organization, internal architecture, hydration-dependent behavior, and spatial distribution of components, all of which may influence their tissue interaction patterns after administration [17]. Environmental scanning electron microscopy has emerged as a useful tool for evaluating the microstructure of hydrated biomaterials while minimizing preparation-induced artifacts, thereby allowing more realistic characterization of formulation architecture and structural organization [11,18]. Therefore, combining ultrastructural characterization with *in vivo* imaging, histological, and molecular analyses may provide broader insight into how different formulations are associated with extracellular matrix remodeling-related responses [8,19].

Recent studies have suggested that biomaterial-associated tissue responses may involve differential modulation of collagen-related molecular pathways and extracellular matrix organization rather than a single uniform reparative mechanism [8,11]. In this context, transepidermal delivery of calcium hydroxyapatite via microneedling has been associated with distinct collagen-related transcriptional responses during experimental dermal remodeling, supporting the concept that different biomaterial formulations may induce distinct matrix-related response patterns [11]. These findings support the rationale for comparative studies investigating whether different injectable bioregenerative formulations are associated with specific structural, imaging-based, histological, and molecular extracellular matrix responses.

Accordingly, this study aimed to comparatively evaluate the ultrastructural characteristics and collagen-related tissue responses associated with commercially available PLLA-, HA-, and PDRN-containing formulations, used alone or in combination, in a murine model of dermal remodeling. The formulation microstructure was characterized by environmental scanning electron microscopy, and tissue response patterns were evaluated by ultrasonography, histology, and RT-qPCR analysis of collagen-related genes. By integrating structural, imaging-based, histological, and molecular endpoints, this study sought to investigate whether different commercially available formulations are associated with distinct dermal remodeling-related responses under the evaluated experimental conditions.

## 2. Results

### 2.1. Structural Characterization by ESEM

**Figure 1:**
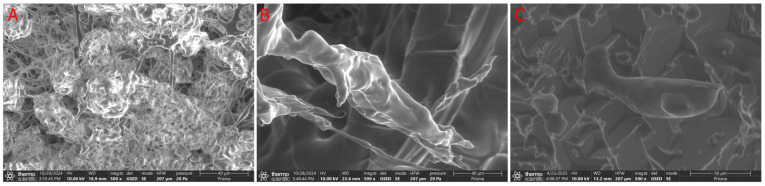
Ultrastructural Characterization of the Individual Biomaterials. Environmental scanning electron microscopy (ESEM) micrographs of the biomaterials used in the study, obtained with a Thermo Fisher Scientific Prisma E microscope. (**A**) Poly-L-lactic acid (PLLA; Rennova), showing irregular microparticles with heterogeneous surface features; (**B**) hyaluronic acid (HA; Perfectha^®^ SubSkin), exhibiting a continuous and compact hydrogel-like morphology; and (**C**) polydeoxyribonucleotide (PDRN; Victa Laboratory), showing an irregular and heterogeneous surface pattern.

Environmental scanning electron microscopy (ESEM) revealed distinct morphological features for each biomaterial (Figure 1A–C). PLLA consisted of irregular microparticles ranging from approximately 10 to 200 µm in diameter, with a predominantly porous and heterogeneous surface (Figure 1A). HA exhibited a morphology consistent with a cross-linked hydrophilic polymer, characterized by a homogeneous three-dimensional network of interwoven structures forming a continuous matrix (Figure 1B). PDRN showed an irregular fibrillar and filamentous arrangement, with heterogeneous clusters of variable density distributed throughout the sample (Figure 1C).

**Figure 2:**
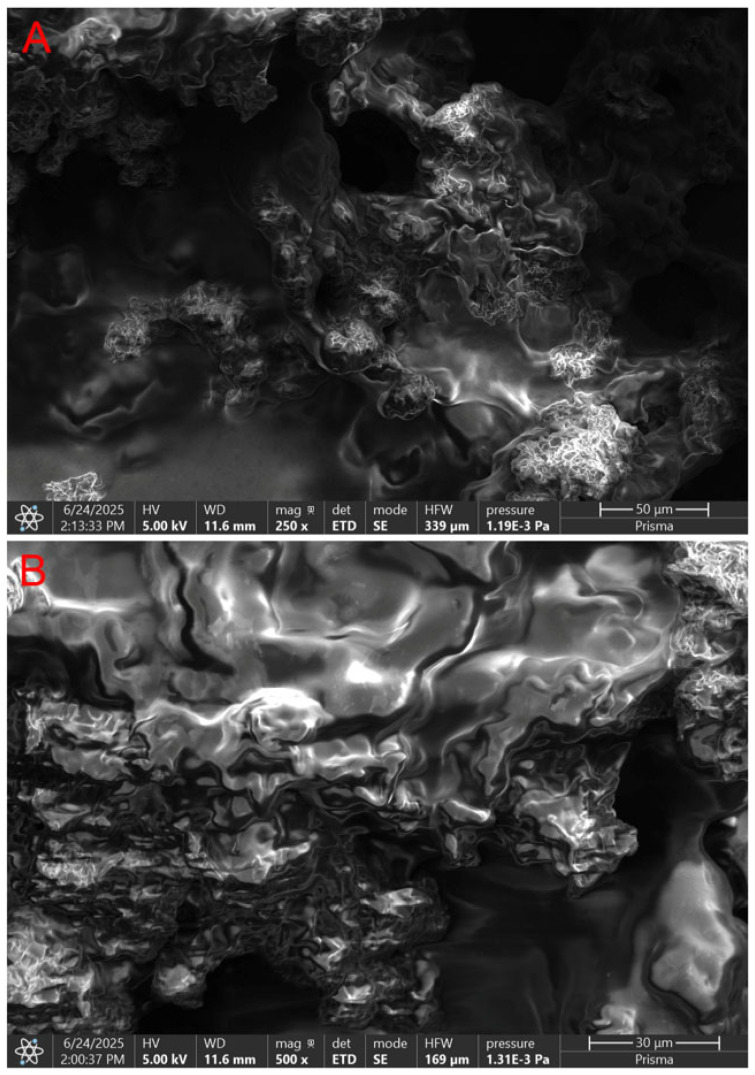
Ultrastructural Characterization of the Combined PLLA, HA, and PDRN Formulation. Environmental scanning electron microscopy (ESEM) micrographs of the combined PLLA, HA, and PDRN formulation. (**A**) At 250× magnification, the formulation exhibits a porous and structurally heterogeneous surface with irregular interconnected domains. (**B**) At 500× magnification, a more evident three-dimensional multiphase microarchitecture is observed, with features consistent with close physical association among PLLA, HA, and PDRN components.

ESEM analysis of the combined PLLA, HA and PDRN formulation demonstrated a homogeneous three-dimensional multiphase architecture (Figure 2A,B). HA formed a continuous matrix with an undulating and relatively uniform surface, within which PLLA microparticles were irregularly dispersed. These particles maintained their characteristic heterogeneous shape and porous surface. Delicate fibrillar structures compatible with PDRN were observed between the PLLA particles and the HA matrix, showing close association with both components. Overall, the combined formulation exhibited a porous and interconnected multiphase structure, suggesting close physical association among the formulation components (Figure 2A,B).

### 2.2. Ultrasound Evaluation

**Figure 3:**
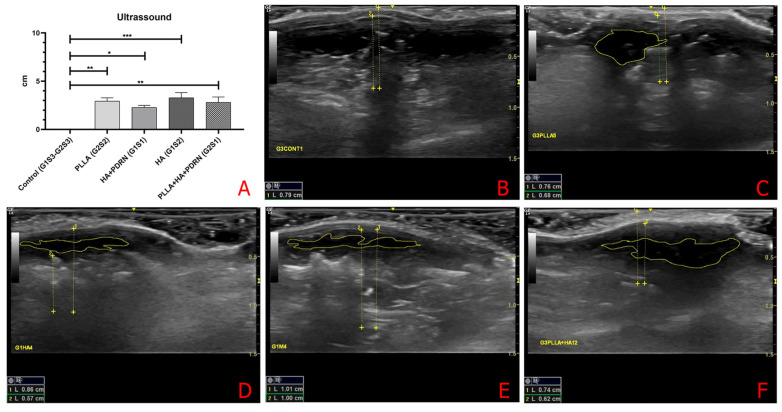
Ultrasound-Based Assessment of Dermal Hypoechogenicity. High-frequency ultrasound evaluation of dermal hypoechogenicity after treatment. Images were acquired using a Logiq E R7 system (GE Healthcare, Chicago, IL, USA). Quantitative measurements were performed using ImageJ^®^ software (version 1.54g, National Institutes of Health, Bethesda, MD, USA), and statistical analysis was conducted using GraphPad Prism version 9.0 (GraphPad Software, San Diego, CA, USA). (**A**) Quantitative analysis of dermal hypoechogenicity showing significant differences among treated groups and the untreated control. (**B**) Untreated control skin (G1S3/G2S3). (**C**) PLLA-treated skin (G2S2). (**D**) HA and PDRN-treated skin (G1S1). (**E**) HA-treated skin (G1S2). (**F**) Skin treated with the combined PLLA, HA, and PDRN formulation (G2S1). Asterisks indicate statistically significant differences (* *p* < 0.05; ** *p* < 0.01; *** *p* < 0.001), according to the comparisons shown in panel A.

Ultrasound evaluation revealed significant differences in hypoechoic signal intensity among the experimental groups (Figure 3A). All treated groups showed higher mean hypoechogenicity values than the control groups (G1S3 and G2S3), although with different magnitudes (Figure 3B). The isolated HA group (G1S2) showed the highest mean hypoechogenicity values (*p* < 0.01), followed by the HA and PDRN (G1S1) groups and the PLLA, HA and PDRN (G2S1) combination, both of which were also significantly higher than controls (*p* < 0.01) (Figure 3D–F). The PLLA-only group (G2S2) showed lower values than the isolated HA group but remained significantly associated with changes in ultrasound echogenicity patterns higher than controls (*p* < 0.05) (Figure 3C). As expected, the control groups showed the lowest hypoechogenicity values, consistent with the absence of injected material (Figure 3B).

Longitudinal body weight analysis showed no statistically significant differences among the control groups, isolated PLLA, HA and PDRN combination, isolated HA, and PLLA, HA, and PDRN combination over 30 days follow-up period (Kruskal–Wallis, *p* > 0.05; Supplementary Figure S1). Mean body weight remained stable throughout the experimental period in all groups, with no evidence of overt systemic effects associated with the interventions.

These ultrasound findings should be interpreted as imaging-based echogenicity changes and not as direct evidence of collagen deposition or definitive dermal remodeling.

### 2.3. Histological Analysis

**Figure 4:**
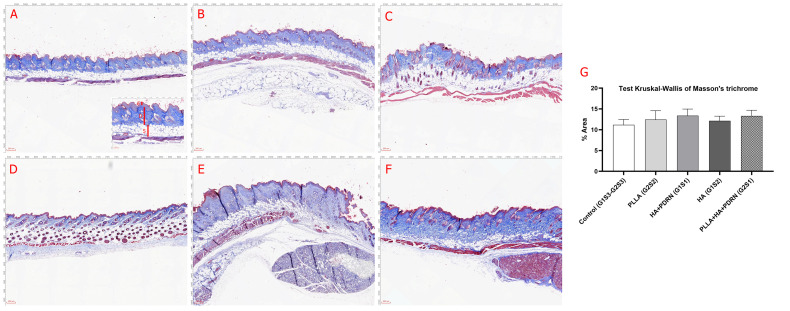
Histological and Morphometric Analysis of Dermal Collagen. Histological and morphometric analysis of dermal collagen after treatment using Masson’s trichrome staining. In Masson’s trichrome staining with aniline blue, collagen fibers are stained blue, whereas other tissue components are counterstained in red/pink tones. Morphometric quantification of collagen-stained area was performed using FIJI (Fiji Is Just ImageJ, version 1.54g; National Institutes of Health, Bethesda, MD, USA) with Otsu thresholding for collagen segmentation, and statistical analysis was conducted using GraphPad Prism version 9.0 (GraphPad Software, San Diego, CA, USA). Representative histological sections are shown for (**A**) untreated control skin from Group 1, The inset in panel A illustrates the histological stratification of the skin: E, epidermis; D, dermis; and S, subcutaneous; (**B**) untreated control skin from Group 2; (**C**) skin from Group 1 treated with HA and PDRN; (**D**) skin from Group 1 treated with HA alone; (**E**) skin from Group 2 treated with the combined PLLA, HA, and PDRN formulation; and (**F**) skin from Group 2 treated with PLLA alone. (**G**) Quantitative comparison of collagen-stained area. Data are presented as mean ± standard deviation. No statistically significant differences were observed among groups (Kruskal–Wallis test, *p* > 0.05). S3 denotes untreated control skin in both groups; in Group 1, S1 and S2 correspond to HA and PDRN and HA alone, respectively; in Group 2, S1 and S2 correspond to the combined PLLA, HA, and PDRN formulation and PLLA alone, respectively.

Masson’s trichrome staining of dorsal skin sections showed preserved tissue architecture across all treatment groups (Figure 4A–G). Control samples from both experimental groups exhibited normal epidermal and dermal organization, with continuous collagen bundles in the papillary and reticular dermis. A similar pattern was observed in animals treated with HA and PDRN and HA alone, with no evidence of epidermal disruption, dermal disorganization, or marked inflammatory infiltrates. In animals receiving PLLA-containing formulations (a combination of PLLA, HA and PDRN or PLLA alone), collagen fibers remained regularly arranged. They were interspersed with residual biomaterial deposits, without signs of necrosis or pronounced inflammatory response. These qualitative findings were consistent with the morphometric analysis of collagen area, which showed no statistically significant differences among groups (Figure 4G).

### 2.4. RT-qPCR Analysis

**Figure 5:**
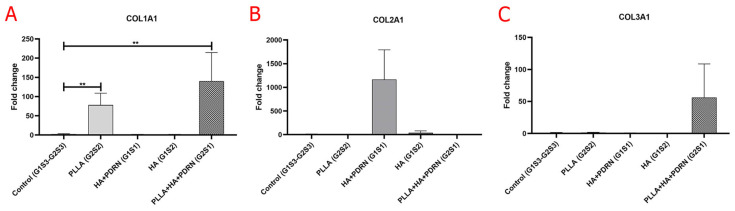
Relative Expression of Collagen-Related Genes. Relative gene expression analysis of collagen-related markers by RT-qPCR. Reactions were performed using a QuantStudio™ 6 Flex Real-Time PCR System (Applied Biosystems™, Foster City, CA, USA) with TaqMan^®^ assays (Thermo Fisher Scientific, Waltham, MA, USA). (**A**) COL1A1 (type I collagen): significantly higher expression was observed in the PLLA-only group (G2S2) and in the combined PLLA, HA, and PDRN formulation group (G2S1) compared with controls, indicating selective upregulation of COL1A1 in PLLA-containing treatments. (**B**) COL2A1 (type II collagen): no statistically significant differences were observed among groups, although the HA and PDRN group (G1S1) showed numerically higher mean values with substantial intragroup variability. (**C**) COL3A1 (type III collagen): the combined PLLA, HA, and PDRN formulation group (G2S1) showed numerically higher expression, but no statistically significant differences were observed among groups. Relative expression was calculated using the 2^−ΔΔCt^ method, with the untreated control group as calibrator. Data are presented as mean ± standard deviation. ** *p* < 0.01 indicates a statistically significant difference between the indicated groups.

Despite no significant differences in histological collagen area among groups, RT-qPCR analysis revealed distinct gene expression patterns after 30 days of treatment (Figure 5A–C). For COL1A1, significantly higher expression was observed in the PLLA-only group and in the PLLA, HA and PDRN group compared with controls (*p* < 0.05) (Figure 5A). Although the PLLA, HA, and PDRN combination group showed numerically higher mean COL1A1 values than the PLLA-only group, this difference was not statistically significant.

No statistically significant differences were observed among groups for COL2A1 expression, although the HA and PDRN group showed a numerically higher mean value with substantial intragroup variability (Figure 5B). Similarly, COL3A1 expression was higher in the group with the combination of PLLA, HA, and PDRN, but this increase did not reach statistical significance (Figure 5C).

Overall, these findings indicate that PLLA-containing formulations were associated with selective upregulation of COL1A1, whereas no significant modulation of COL2A1 or COL3A1 was observed under the conditions evaluated.

## 3. Discussion

The most relevant finding of this study was the selective increase in COL1A1 expression in the PLLA and combination PLLA, HA and PDRN groups after 30 days, indicating that PLLA-containing formulations were associated with higher COL1A1 expression than the other evaluated conditions at the 30-day endpoint. Increased COL1A1 expression may reflect an early transcriptional response associated with extracellular matrix remodeling rather than mature collagen deposition detectable at the tissue level within the evaluated experimental period. This pattern is biologically coherent, since type I collagen predominates during the maturation phase of tissue repair and reflects matrix consolidation rather than early provisional deposition [10,20]. In this context, these findings are consistent with the expected late collagen-related response reported for formulations containing PLLA, favoring structural extracellular matrix reorganization rather than an early transient response. This interpretation is consistent with previous evidence showing that lactic acid–based biostimulators can promote gradual fibroblast activation and neocollagenesis over time, with more evident molecular or structural effects at later observation points [14,21]. Notably, significant COL1A1 upregulation was observed in both PLLA-containing formulations, namely the PLLA-only formulation and the combined PLLA, HA, and PDRN formulation. This finding suggests an association between PLLA-containing formulations and collagen-related transcriptional responses in this model, without allowing definitive attribution of the effect to PLLA as an isolated component. Although the combined formulation also showed COL1A1 induction, the absence of clear histological superiority over isolated PLLA suggests that the combination does not necessarily translate into greater short-term tissue-level effect at a single 30-day endpoint.

Importantly, increased COL1A1 expression should not be interpreted as direct evidence of mature collagen accumulation. Transcriptional activation and histologically detectable collagen deposition represent distinct biological levels and may occur at different time points during extracellular matrix remodeling. Therefore, the absence of a significant increase in collagen-stained area does not necessarily contradict the RT-qPCR findings. Instead, COL1A1 upregulation may reflect an early or intermediate molecular response that precedes detectable extracellular matrix accumulation at the tissue level. Additional protein-level analyses, such as immunohistochemistry or Western blotting, and longer follow-up periods would be required to confirm whether these transcriptional changes translate into mature collagen deposition.

The absence of significant differences in COL3A1 is also relevant and should not be interpreted as a lack of biological activity. Type III collagen is typically associated with the early proliferative phase of repair and is progressively reduced as remodeling advances and type I collagen becomes predominant [20]. Therefore, the absence of significant COL3A1 upregulation at the 30-day endpoint is consistent with the interpretation that the evaluated period may have captured a later stage of extracellular matrix remodeling, in which type I collagen transcription becomes more prominent than type III collagen expression. Based on previous evidence regarding the temporal dynamics of collagen remodeling, longer follow-up periods may be necessary to determine whether these formulations, particularly when used in combination, are associated with subsequent modulation of other collagen subtypes. Further studies evaluating additional experimental time points are therefore required to better characterize the temporal profile of collagen subtype expression induced by these formulations. Likewise, COL2A1 did not show significant modulation under the evaluated conditions, which is consistent with its limited role as a major component of adult dermal extracellular matrix.

Histological preservation across all groups, without evidence of exacerbated inflammation or tissue disruption, supports the short-term biocompatibility of the evaluated formulations. This is particularly important for PLLA-containing formulations, since their biological activity depends on controlled tissue stimulation without excessive inflammatory burden. Previous reports evaluating PLLA-containing biostimulators have described mild and self-limited tissue responses associated with fibroblast activation and extracellular matrix remodeling [14,22]. In the present model, the absence of marked histological alterations at 30 days is therefore compatible with a regulated remodeling process rather than biological inactivity.

The combined formulation deserves cautious interpretation. Although PLLA, HA, and PDRN exhibited an integrated microstructural organization and significant COL1A1 upregulation, the present design does not allow a definitive conclusion regarding synergistic interaction among components. It is plausible that each biomaterial contributes through partially distinct mechanisms: PLLA as a delayed collagen biostimulator, HA as a matrix-organizing and hydration-supporting component, and PDRN as a potential modulator of early repair signaling, angiogenesis, or cellular recovery [15,16,23]. However, because only a single endpoint was evaluated, the temporal complementarity of these effects remains hypothetical and should be interpreted conservatively.

The ultrasound findings also support the concept that formulation behavior is not determined solely by biochemical composition. Although ultrasound imaging may provide indirect information regarding tissue organization and local structural changes, echogenicity alterations alone do not constitute definitive evidence of dermal remodeling or collagen deposition. By physical organization and tissue interactions. The greater apparent retention observed with isolated PDRN, compared with the more dispersed profiles in PLLA-containing or HA-containing conditions, suggests that different formulations may behave differently after administration, even when overt histological changes are not yet evident. Although these observations are mechanistic and exploratory, they reinforce the importance of considering formulation architecture as a relevant determinant of *in vivo* behavior, especially in studies of injectable or biobioregenerative formulations.

This study has limitations. The analysis was restricted to a single 30-day time point, which limits the ability to characterize early inflammatory and proliferative events, particularly those potentially associated with PDRN. In addition, although the split-site design reduced biological variability and followed a reduction strategy, site-level observations were not fully independent. Finally, the molecular panel was focused on collagen-related targets and did not include inflammatory, angiogenic, or matrix-remodeling mediators that could better explain early biological differences among formulations.

Taken together, the present findings indicate that PLLA-containing formulations are more consistently associated with late collagen remodeling than isolated PDRN- or HA-containing conditions in this model. Rather than demonstrating broad superiority of combined formulations, the data support a more nuanced interpretation in which PLLA appears to be the principal determinant of COL1A1 induction at 30 days, while the contribution of HA and PDRN may depend on earlier or complementary phases of tissue repair. This interpretation is biologically plausible, consistent with the current literature, and relevant for the rational design of future combinatorial bioregenerative strategies.

## 4. Materials and Methods

### 4.1. Biomaterials and Injectable Formulations

Hyaluronic acid (HA) gel (Perfectha^®^ SubSkin, 20 mg/mL; Sinclair France SAS, Lyon, France; ANVISA registration no. 81277680003; batch no. 230221-3). Poly-L-lactic acid (PLLA) (Rennova^®^ Elleva 210 mg; Rennova, Goiânia, GO, Brazil; ANVISA registration no. 80451960236). Polydeoxyribonucleotide (PDRN) 0.5% (2 mL; Victa Laboratório de Manipulação, São Paulo, SP, Brazil), associated with growth factors (TGF-β, IGF-1, EGF, bFGF), vitamin B3 (niacinamide), and catalase, according to the manufacturer’s formulation. All products were used within the manufacturer-recommended expiration dates.

### 4.2. Preparation of Hydrogels Containing PLLA, HA, and PDRN

The hydrogels were prepared in two formulations: PLLA associated with HA and the combination of PLLA, HA, and PDRN. In the primary formulation (PLLA and HA), the PLLA was initially reconstituted according to the manufacturer’s instructions using the Rennova^®^ Mixer device under aseptic conditions at controlled room temperature (22–25 °C), ensuring adequate dispersion of the microparticles. The reconstituted PLLA was transferred to a sterile syringe and combined with the HA-containing syringe in a 1:1 ratio. The mixing was performed by manually exchanging the contents between the syringes coupled by a sterile Luer-lock connector, with successive passes until a visually homogeneous mixture was obtained.

In the PLLA, HA and PDRN formulation, the base mixture of PLLA and HA was initially prepared as described previously. Then, PDRN was added to the already homogenized mixture, and a new exchange procedure was performed in a closed syringe system until complete integration of the components.

Throughout the process, no external heat sources were applied, thereby avoiding potential alterations in the materials’ physicochemical properties. The formulations were stored under refrigeration (4 °C), protected from light, and used within the recommended stability period. Before experimental application, the hydrogels were inspected for signs of degradation or physicochemical changes, such as precipitation, phase separation, or changes in viscosity.

Throughout the process, all batches of materials were properly recorded and labeled, in accordance with the traceability protocols required by current legislation and good manufacturing practices. Controlling the proportions and standardizing the mixing process ensured the production of homogeneous, stable hydrogels suitable for subsequent stages of the study, thereby allowing the preparation of reproducible, reliable formulations.

### 4.3. Structural Characterization of Hydrogels

The bioregenerator samples were imaged directly using an Environmental Scanning Electron Microscope (ESEM) using a Prism E-SEM (Thermo Fisher Scientific, Waltham, MA, USA), with a thermionic tungsten filament source. The samples were deposited on a support previously cooled to −5 °C. The SEM images were captured using a gaseous secondary electron detector (GSED) at 10 kV, under a water vapor atmosphere, with a spot size of 4.0 µm, pressure 8 × 10^3^.

For the analysis of the combined samples, the high-vacuum mode with the Everhart-Thornley detector (ETD), which is well suited for capturing secondary electrons, was used. This mode is essential for minimizing electron scattering and ensuring superior image resolution. The microscope was operated at 30 kV, enabling real-time image acquisition and precise focus and contrast adjustments. The micrographs obtained were evaluated for sample topography, including roughness and porosity, providing relevant information on morphology.

### 4.4. Sample Calculation and Animal Models

Sample size was estimated a priori based on the primary outcome, relative COL1A1 gene expression, and the comparison between treated and control sites. A two-sided significance level of α = 0.05 and a statistical power of 80% (β = 0.20) were adopted. Based on previous literature and the research group’s experience, a coefficient of variation of approximately 20% was assumed for the main outcome measures, and a minimum biologically relevant difference of 30% between groups was defined [24]. Under these assumptions, the minimum required sample size for a two-sample comparison of means was estimated as 7 animals per experimental group. Although the present study used a split-site intra-animal design, the animal was considered the primary experimental unit for sample size planning and interpretation, in accordance with the 3Rs principle and published recommendations for animal experimentation [24]. A total of 14 male ad libitum mice (Swiss strain), aged 28–38 weeks and weighing approximately 43 g, were included and allocated into two experimental groups (G1, *n* = 7; G2, *n* = 7). The animals were obtained from the Animal Breeding and Experimentation Facility of the Institute of Health Research (IPS) at the Universidade Estadual de Montes Claros (UNIMONTES), Brazil. The experimental protocol was approved by the Animal Ethics Committee of Universidade Estadual de Montes Claros (CEUA/UNIMONTES; protocol no. 003/2024, approved on 12 April 2024). Animals were housed in standard polypropylene cages under controlled environmental conditions (22 ± 2 °C, 12 h light/dark cycle), with free access to chow and water *ad libitum*, and underwent a 7-day acclimatization period before the procedures.

The experimental design followed a split-site intra-animal model. In each animal, three standardized dorsal treatment sites were defined, resulting in a total of 42 treated or control sites across all animals. In Group 1 (G1), site 1 received HA and PDRN, site 2 received isolated PDRN, and site 3 received no treatment (control). In Group 2 (G2), site 1 received the combination of PLLA, HA, and PDRN site 2 received isolated PLLA, and site 3 received no treatment (control). Control sites did not receive any injection or vehicle administration. Treatment sites were assigned to fixed anatomical dorsal positions in all animals to standardize application depth, local tissue exposure, imaging acquisition, and tissue harvesting. Thus, randomization was applied to animal allocation between experimental groups, whereas treatment-site position within each group was standardized and not randomized.

Animals were individually identified throughout the study, and the dorsal region was shaved before treatment and every two days thereafter until euthanasia to maintain clear visualization of the intervention sites. No animal losses occurred during the experimental period. Since sample size estimation was driven by the primary molecular endpoint (COL1A1 expression), the study may be underpowered for secondary histological, imaging-based, and ultrastructural outcomes; therefore, these analyses were interpreted in an exploratory and descriptive manner.

### 4.5. In Vivo Study

The animals used in the experiment were initially anesthetized with a combination of ketamine hydrochloride (100 mg/kg/animal; Ketamine Agener^®^, Agener União, Brazil) and xylazine hydrochloride (10 mg/kg/animal; Calmium^®^, Agener União, Brazil), with specific doses of 0.03 mL of xylazine and 0.05 mL of ketamine per animal. Subsequently, trichotomy was performed on the dorsal region. The treatments were administered intradermally into the three previously standardized dorsal sites for each animal, using a 22G microcannula and 0.5 mL per site for each formulation (described in Supplementary Table S1). All procedures were conducted in accordance with Protocol 003/2024, which the UNIMONTES Animal Use Ethics Committee had approved. Animals were monitored daily throughout the experimental period for general health status, mobility, grooming behavior, food and water intake, and signs of pain or distress. No additional postoperative analgesia was administered, as the procedure was considered minimally invasive and was performed under appropriate anesthesia. No humane endpoint criteria were reached. All animal procedures and reporting were conducted in accordance with ARRIVE 2.0 recommendations.

### 4.6. Ultrasound

After 30 days of treatment with bioregenerative agents, the animals were anesthetized with a combination of ketamine hydrochloride (100 mg/kg per animal; Ketamine Agener^®^, Agener União, Brazil) and xylazine hydrochloride (10 mg/kg per animal; Calmium^®^, Agener União, Brazil), and subsequently subjected to ultrasound examination of the dorsal region at both treatment application sites using a high-frequency ultrasound system (Logiq E R7, GE Healthcare, Chicago, IL, USA). Following imaging, the animals were euthanized by an overdose of the same anesthetic agents, combined with a lethal intracardiac injection of propofol (0.4 mL). All animal experiments were conducted in full compliance with the ARRIVE guidelines, ensuring high standards of scientific rigor and ethical responsibility. All procedures were approved by the Animal Ethics Committee of Universidade Estadual de Montes Claros (CEUA/UNIMONTES; protocol no. 003/2024).

Quantitative measurements of dermal, epidermal, and subcutaneous tissue thickness were obtained from ultrasound images using a standardized methodological approach. This protocol was based on previously described ultrasound-based tissue assessment procedures [25]. The acquired images were analyzed after calibration of the spatial scale according to the equipment reference. Subsequently, the region corresponding to the injected treatment site was manually delineated in each image, after calibrating the spatial scale according to the equipment’s reference. Tissue thickness measurements were performed using the linear measurement tool in ImageJ^®^ (version 1.54g, National Institutes of Health, Bethesda, MD, USA), with standardized analysis criteria across all samples. The values obtained were organized and analyzed statistically using GraphPad Prism version 9.0 (GraphPad Software, San Diego, CA, USA). All outcome assessments were performed under total blinding. Investigators responsible for ultrasound image interpretation, histological evaluation, morphometric analysis, and molecular analyses were blinded to group allocation and treatment-site identity during data acquisition and analysis.

### 4.7. Histological Analysis

Following euthanasia, dorsal skin samples were carefully harvested for subsequent histological and morphometric analysis. Histological techniques are paramount for the detailed microstructural assessment of biological tissues, providing critical insights into their composition, organization, and pathological alterations at various magnifications. Specifically, three skin samples, approximately 3 mm thick, were collected from the treated dorsal region of each mouse to investigate collagen fiber production and architecture. The collected specimens were fixed immediately in a 10% (*v*/*v*) neutral buffered formalin solution and transferred to 15 mL Falcon tubes for a fixation period of 72 h. Subsequently, samples underwent conventional histological processing for paraffin embedding. To enable detailed visualization and quantification of collagen fibers, the paraffin sections were stained with the Masson’s Trichrome kit containing aniline blue (Suetam^®^, lot B2401), strictly following the manufacturer’s instructions [26,27,28]. The stained slides were digitally scanned using a slide scanner (MoticEasyScan Infinity 60, Motic, Hong Kong, China). This comprehensive approach permits a reliable evaluation of collagen density and fiber organization within the treated dermal tissues.

Morphometric analysis was performed by quantifying the percentage of area occupied by collagen using FIJI (Fiji Is Just ImageJ, version 1.54g) [29], an open-source distribution of ImageJ (version 1.54g, National Institutes of Health, Bethesda, MD, USA) with pre-installed plug-ins. The images were previously calibrated for spatial scale, and quantification was performed by color segmentation with threshold definition using a specific Otsu plug-in to identify areas stained blue (collagen) in Masson’s Trichrome [28]. The percentage of collagen area was calculated relative to the total area of the analyzed field, while maintaining standardized brightness, contrast, and thresholding parameters across all samples to ensure reproducibility and comparability between experimental groups. The collagen area percentage values obtained through analysis in FIJI (Fiji Is Just ImageJ, version 1.54g) were organized and subjected to statistical analysis using GraphPad Prism version 9.0 (GraphPad Software, San Diego, CA, USA).

### 4.8. Euthanasia of Animals for Subsequent qPCR Analysis

After treatment of the mice with collagen biostimulators, the animals were euthanized, fully following the ethical protocols established by the Ethics Committee. Subsequently, tissue samples (skin) from the treated area were collected and stored in RNA stabilizer solution, in a freezer at −80 °C, for subsequent analysis.

### 4.9. RNA Extraction

Total RNA was extracted from tissue samples previously macerated in liquid nitrogen using a standardized protocol based on TRIzol^®^ reagent. The procedure followed established guidelines for RNA isolation using phenol–chloroform, extraction including phase separation with chloroform, RNA precipitation with isopropanol, and washing with 75% ethanol [8,11]. The resulting precipitate was resuspended in RNase-free water, and the samples were stored at −80 °C until subsequent analyses.

### 4.10. Quantification and Purity Assessment (Spectrophotometer)

After extraction, the RNA was quantified and analyzed for purity by spectrophotometry using the NanoDrop 2000 equipment (Thermo Fisher Scientific, Waltham, MA, USA). Absorbance ratios A260/A280, indicative of protein contamination, and A260/A230, related to the presence of organic compounds or salts, were considered [30]. The values obtained were recorded and analyzed to verify the suitability of the samples for subsequent steps. They were classified as appropriate or inappropriate according to widely accepted criteria, such as low concentration, evidence of contamination, or material degradation.

### 4.11. cDNA Synthesis (Reverse Transcription)

cDNA synthesis was performed from previously quantified total RNA using a standard reverse transcription protocol. The RNA concentration used per reaction was standardized to a final volume of 7 µL, adjusted with RNase-free water. After this adjustment, specific reverse transcription reagents were added. Treatment was carried out with Buffer 10X (Thermo Fisher Scientific, Waltham, MA, USA), DNase (Thermo Fisher Scientific, Waltham, MA, USA), and EDTA (Thermo Fisher Scientific, Waltham, MA, USA) to inactivate the enzyme. The primer mixture consisted of Oligo dT (Thermo Fisher Scientific, Waltham, MA, USA), random primers (Thermo Fisher Scientific, Waltham, MA, USA), and dNTPs (Promega Corporation, Madison, WI, USA), according to the manufacturer’s recommendations. Subsequently, Mix 2 was added, containing Buffer 5X (Thermo Fisher Scientific, Waltham, MA, USA), DTT (Thermo Fisher Scientific, Waltham, MA, USA), and RNase OUT (Thermo Fisher Scientific, Waltham, MA, USA), used as an RNase inhibitor. Next, the reverse transcriptase enzyme M-MLV RT (Thermo Fisher Scientific, Waltham, MA, USA) was added.

The reactions were conducted in an Eppendorf Mastercycler thermocycler (Eppendorf, Hamburg, Germany) with appropriate incubation steps for denaturation, primer annealing, cDNA synthesis, and enzyme inactivation. At the end of the reaction, the reverse transcription products were stored at −20 °C until their use in gene expression analyses by RT-qPCR (QuantStudio™ 6 Flex Real-Time PCR system, Applied Biosystems™, Foster City, CA, USA).

### 4.12. Real-Time Polymerase Chain Reaction (RT-qPCR)

Gene expression quantification was performed by real-time PCR (qPCR) using the QuantStudio™ 6 Flex Real-Time PCR system (Applied Biosystems™, Foster City, CA, USA) and TaqMan^®^ probes (Thermo Fisher Scientific, Waltham, MA, USA) in 384-well plates. Previously synthesized cDNA was used to analyze the genes COL1A1 (Mm03950652_s1), COL2A1 (Mm01309565_m1), and COL3A1 (Mm00802300_m1), with the RN18S gene (Mm03928990_m1) as an endogenous control for normalization [8].

The reactions were prepared to a final volume of 10 µL, containing TaqMan™ Universal PCR Master Mix (2X) (Applied Biosystems, Thermo Fisher Scientific, Waltham, MA, USA), TaqMan™ Gene Expression Assay probes (20X) (Thermo Fisher Scientific, Waltham, MA, USA), cDNA, and nuclease-free water, according to the manufacturer’s recommendations. All samples were analyzed in triplicate under standard TaqMan assay cycling conditions.

The amplification protocol consisted of an initial denaturation step, followed by 40 amplification cycles, with temperature and time parameters specific to TaqMan probes. Fluorescence detection was performed automatically by the equipment, allowing real-time monitoring of the amplification. The system software determined Ct (cycle threshold) values. Samples without amplification, with high Ct, or showing inconsistencies between duplicates/triplicates were excluded from the analysis. Differences in sample size across specific molecular comparisons were due to technical losses during sample processing and did not reflect animal loss. Relative gene expression was calculated using the 2^−ΔΔCt^ method, with the control group used as the calibrator for data normalization. Initially, ΔCt values were obtained by subtracting the Ct value of the reference gene (RN18S) from the Ct value of the target gene. Subsequently, ΔΔCt values were determined by subtracting the mean ΔCt of the control group from the ΔCt of each treated sample. Relative gene expression was then expressed as fold change according to the formula 2^−ΔΔCt^.

### 4.13. Experimental Controls Used in Real-Time PCR

To ensure the reliability of qPCR results, appropriate experimental controls were included. No Template Control (NTC) reactions, prepared without the addition of cDNA, were used to monitor possible contaminations or nonspecific amplifications. The absence of amplification in these reactions confirmed the integrity of the reagents and experimental conditions. In addition, biological controls consisted of untreated sites (no injection or vehicle administration) collected within the same experimental design, which served as the reference for comparative analysis of gene expression. This control condition was used as the calibrator in the calculation of relative expression by the ΔΔCt method.

### 4.14. Statistical Analysis

The statistical analysis of the data was performed using GraphPad Prism^®^ software, version 9.0 (GraphPad Software, San Diego, CA, USA). Initially, the normality of the data distribution was assessed using the Kolmogorov–Smirnov test. For normally distributed variables, comparisons between experimental groups were conducted using one-way analysis of variance (ANOVA), followed by Tukey’s post hoc test for multiple comparisons. For data that did not follow a normal distribution, non-parametric tests were used, including the Kruskal–Wallis test for multiple-group comparisons and the Mann–Whitney test for two independent groups. Categorical variables were analyzed using the chi-square test or Fisher’s exact test, as appropriate for the sample characteristics and expected frequencies in the categories. When applicable, binary logistic regression was used to estimate the odds ratio between the evaluated groups. The results were expressed as mean ± standard deviation for parametric data or as median and interquartile range for non-parametric data. In all analyses, a significance level of 5% (*p* < 0.05) was adopted. These statistical procedures were employed to ensure methodological rigor and analytical reliability of the experimental findings [24]. Because multiple treatment sites were evaluated within the same animal, observations were not fully independent. The split-site design reduced biological variability and enabled intra-animal comparison; however, no formal mixed-effects model was applied, which should be considered when interpreting the results. The manuscript was revised to explicitly comply with ARRIVE 2.0 recommendations, including reporting of the experimental unit, allocation strategy, housing conditions, blinding, animal welfare monitoring, and primary outcome definition.

## 5. Conclusions

Within the limitations of this murine study, PLLA-containing formulations, used alone or in combination with HA and PDRN, were more consistently associated with COL1A1 upregulation after 30 days, suggesting a stronger association with late dermal remodeling. Histological preservation across all groups supports the short-term biocompatibility of the evaluated biomaterials. At the same time, differences in formulation architecture and ultrasound behavior suggest that physical organization may influence *in vivo* tissue interaction. Rather than demonstrating clear superiority of combined formulations, the findings indicate that PLLA was the main determinant of the collagen-related molecular response at this time point. These results support the rational development of PLLA-based and combinatorial bioregenerative strategies for dermal remodeling.

## Data Availability

The data presented in this study are available from the corresponding author upon reasonable request.

## Supplementary Materials

The following supporting information can be downloaded at: https://www.mdpi.com/article/10.3390/ijms27114936/s1.
