# Supplementary material for: Comparative ESEM Characterization and Collagen-Related Tissue Responses to Commercial Injectable Bioregenerative Formulations in a Murine Model
Source: Int J Mol Sci. 2026 May 29;27(11):4936. doi: 10.3390/ijms27114936 (PMC13256218; doi:10.3390/ijms27114936)
Supplement: Supplementary file 1 [file ijms-27-04936-s001.zip › ijms-4305828-supplementary.pdf]

Supplementary Materials

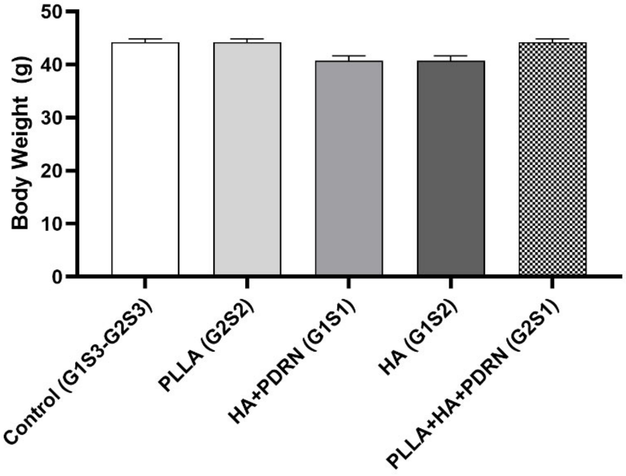

Supplementary Figure S1. Final Body Weight After 30-Day Treatment. Final body weight of mice after 30 days of treatment with the experimental formulations. Bars represent the mean body weight (g) for each group: untreated control (G1S3/G2S3), PLLA alone (G2S2), HA and PDRN (G1S1), HA alone (G1S2), and the combined PLLA, HA, and PDRN formulation (G2S1). Error bars indicate standard deviation.

Table 1. Treatments.

**Treatments**	**Composition**	**Final Volume Applied (mL)**
HA + Water for injection	1.5 mL and HA + 8.5 mL and Water for injection	0.5
HA + PDRN + Water for injection	1.5 mL and HA + 1.5 mL and PDRN + 7 mL and Water for injection	0.5
PLLA + Water for injection	7 mL and PLLA + 3 mL and Water for injection	0.5
PLLA + HA + PDRN	7 mL PLLA + 1.5 mL and HA + 1.5 mL de PDRN	0.5

Supplementary Table S1. Composition and Final Volume of the Administered Formulations. Composition and final administered volume of each experimental formulation used in the study. All treatments were prepared according to the predefined formulation protocol and administered at a final volume of 0.5 mL per application. The table details the component distribution for PLLA alone, HA and PDRN, HA alone, and the combined PLLA, HA, and PDRN formulation.
